# 奥布替尼联合R-CHOP方案治疗初治高危伴结外累及的非生发中心起源的弥漫大B细胞淋巴瘤的临床研究

**DOI:** 10.3760/cma.j.cn121090-20240914-00347

**Published:** 2025-02

**Authors:** 宝平 郭, 明月 王, 成成 廖, 达 周, 晴 柯, 喆 李, 洪 岑

**Affiliations:** 广西医科大学附属肿瘤医院淋巴血液肿瘤科，南宁 530021 Department of Hematology, Guangxi Medical University Cancer Hospital, Nanning 530021, China

**Keywords:** 淋巴瘤，大B细胞，弥漫性, 非生发中心起源, 国际预后指数高危, 结外病变, 奥布替尼, Lymphoma, large B-Cell, diffuse, non-GCB, High-risk IPI, Extranodal disease, Orelabrutinib

## Abstract

**目的:**

探索奥布替尼与R-CHOP方案联合使用，治疗高危伴结外累及的非生发中心起源的弥漫大B细胞淋巴瘤（non-GCB DLBCL）患者的疗效与安全性。

**方法:**

回顾性纳入2021年1月至2022年12月期间广西医科大学附属肿瘤医院淋巴血液肿瘤科收治的DLBCL患者35例。患者为经免疫组化证实、国际预后指数评分为3～5分，且经PET-CT证实存在2处或以上结外受累的non-GCB DLBCL。治疗方案为标准R-CHOP方案联合奥布替尼口服（150 mg/d），完成6个周期治疗。对于治疗中出现中性粒细胞缺乏症或3级中性粒细胞减少伴有发热的患者，下一个周期开始在化疗结束48 h后给予预防性聚乙二醇化粒细胞集落刺激因子治疗。观测终点包括有效率（ORR）、完全缓解（CR）率、无进展生存（PFS）期、总生存（OS）期和安全性评估。

**结果:**

患者中位年龄53（21～72）岁，中位随访时间为28（12～36）个月，其中19例为双表达患者。ORR为88.6％，CR率为68.6％，2年PFS率和OS率分别为68.6％（95％ *CI* 54.0％～87.2％）和87.5％（95％ *CI* 76.7％～100％）。双表达DLBCL患者的2年PFS率低于非双表达患者［54.4％（95％ *CI* 35.4％～84.2％）对85.2％（95％ *CI* 68.3％～100％），*P*＝0.048］。严重不良事件包括发热性中性粒细胞减少症、肺炎和心房扑动，未观察到治疗相关死亡病例。

**结论:**

在高危伴有结外受累的non-GCB DLBCL患者中，奥布替尼联合R-CHOP方案治疗显示出较好的疗效和可控的不良反应。

## Abstract (en)

**Objective:**

To explore the efficacy and safety of orelabrutinib combined with R-CHOP in patients with high-risk nongerminal center B-cell（non-GCB）diffuse large B-cell lymphoma（DLBCL）with extranodal involvement.

**Methods:**

This retrospective study was conducted on 35 patients who were seen at Guangxi Medical University Cancer Hospital and were immunohistochemically confirmed to have non-GCB DLBCL, had an International Prognostic Index score of 3–5, and confirmed to have ≥2 extranodal involvement on PET/CT. The treatment comprised the standard R-CHOP regimen combined with oral orelabrutinib（150 mg/day）for six cycles. In patients who developed neutropenia or grade 3 neutropenia with fever during treatment, administration of prophylactic pegylated granulocyte colony-stimulating factor 48 h after the end of chemotherapy was started on the next cycle. The endpoints included overall response rate（ORR）, complete response（CR）rate, progression-free survival（PFS）time, overall survival（OS）time, and safety assessment.

**Results:**

The 35 eligible patients enrolled had a median age of 53 years（21–72 years）and a median follow-up time of 28 months（12–36 months）; 19 patients had double-expressor（DE）status. The ORR was 88.6％, and the CR rate was 68.6％. The 2-year PFS and OS rates were 68.6％（95％ *CI* 54.0％–7.2％）and 87.5％（95％ *CI* 76.7％–100％）, respectively. The 2-year PFS rate was significantly lower in patients with DE status than in those without DE status［54.4％（95％ *CI* 35.4％–84.2％）*vs*. 85.2％（95％ *CI* 68.3％–100％）, *P*＝0.048］. Serious adverse events included febrile neutropenia, pneumonia, and atrial flutter, but no treatment-related deaths.

**Conclusion:**

In patients with high-risk non-GCB DLBCL and extranodal involvement, the combination of orelabrutinib with R-CHOP regimen had good efficacy and manageable toxicity.

目前国际预后指数（IPI）、淋巴瘤细胞起源（origin of cells，COO）以及分子亚型等是被广泛应用的弥漫大B细胞淋巴瘤（DLBCL）预后评价指标。其中，高IPI指数和非生发中心起源（non-GCB）亚型被认为是预后不良的重要标志[1]。此外，对于伴有结外累及的患者，其临床表现往往呈现出高侵袭性特征，这部分患者通常是MCD分子亚型，预后极差[2]–[4]。如何有效改善这部分患者的预后，是当前临床实践中亟待解决的主要挑战。

本研究旨在探索奥布替尼联合R-CHOP方案在这类患者中的有效性和安全性，为改善预后不良的DLBCL患者的治疗效果提供新的临床证据和思路。

## 病例与方法

1. 病例：本研究为一项回顾性真实世界研究，纳入了2021年1月至2022年12月期间，在广西医科大学附属肿瘤医院接受奥布替尼联合R-CHOP方案治疗的DLBCL患者。纳入标准：患者年龄达到18岁或以上，经组织学确诊为non-GCB DLBCL，且通过PET-CT证实至少存在2个淋巴结外部位的受累，同时IPI评分为3～5分。免疫组化提示MYC与BCL2蛋白高表达，称为双表达。排除标准则包括中枢神经系统淋巴瘤、HIV阳性的DLBCL、双打击淋巴瘤以及由惰性淋巴瘤转化而来的DLBCL患者。病历资料不完整或失访的患者被排除在外。本研究已获得广西医科大学附属肿瘤医院伦理委员会的批准（批件号：LW2024091），患者均已知情同意。

2. 治疗方案：患者的治疗方案为奥布替尼联合R-CHOP方案，具体用药包括：静脉注射利妥昔单抗（375 mg/m²）、环磷酰胺（750 mg/m²）、表阿霉素（70 mg/m²）、长春新碱（1.4 mg/m²，最大总剂量限制为2 mg）以及口服泼尼松（100 mg/d，持续5 d）和奥布替尼（150 mg/d）。治疗周期为21 d，重复6个周期。对于在治疗周期中出现粒细胞缺乏或3级中性粒细胞减少伴发热的患者，在后续每一个周期化疗结束后48 h，给予预防性聚乙二醇化粒细胞集落刺激因子（Peg-G-CSF）治疗。根据DLBCL中枢神经系统复发预测模型[5]，对于高危患者采用三联鞘注（甲氨蝶呤12.5 mg、阿糖胞苷35 mg、地塞米松5 mg）预防中枢神经系统复发。本研究未常规进行预防性抗感染治疗。

3. 疗效与安全性评估：采用2014版Lugano淋巴瘤疗效评价标准来评估患者的治疗效果。基线成像检查包括PET-CT，而在4个治疗周期后以及治疗结束时，会再次进行PET-CT检查以评估是否达到代谢缓解。通过住院病历、门诊就诊记录和电话对所有患者进行定期随访，随访间隔为3～6个月，随访截止日期为2023年12月31日。无进展生存（PFS）期定义为从诊断到疾病进展、复发、任何原因导致的死亡或最后一次随访之间的时间；总生存（OS）期定义为从诊断到任何原因导致的死亡或最后一次随访之间的时间。有效率（ORR）为部分缓解（PR）率与完全缓解（CR）率的总和。治疗期间发生的所有不良事件，均按照美国国家癌症研究所不良事件通用术语标准5.0版进行评估。

4. 统计学处理：患者的临床病理特征数据采用描述性统计方法进行总结。计数资料采用频数和百分比（％）表示，组间比较采用卡方检验或Fisher确切概率法。计量资料采用*M*（范围）或*x*±*s*表示，采用Mann-Whitney *U*检验。所有的统计分析均使用SPSS 23.0版本软件完成。采用Kaplan-Meier生存曲线描述PFS和OS。在本研究中，双侧*P*<0.05被认为差异具有统计学意义。

## 结果

1. 入组患者的临床特征：本研究共纳入了35例符合纳入标准的non-GCB DLBCL患者，其详细基线特征参见表1。在诊断时，患者的中位年龄为53（21～72）岁，IPI评分为3～5分，且均通过PET-CT证实存在至少2处结外病灶。值得注意的是，19例（54.3％）患者为双表达DLBCL。这些患者的结外受累部位主要包括浆膜腔、肾脏及肾上腺、胃肠道、乳腺、骨髓以及卵巢。其中20例中枢神经系统复发指数高危的患者进行了三联鞘注预防治疗。

**表1:** 35例非生发中心起源的弥漫大B细胞淋巴瘤患者的临床特征

临床特征	例（％）
性别	
男	16（45.7）
女	19（54.3）
年龄	
<60岁	24（68.6）
≥60岁	11（31.4）
双表达	
是	19（54.3）
否	16（45.7）
结外累及部位	
肾或肾上腺	10（28.6）
肝	2（5.7）
肺	2（5.7）
骨	5（14.3）
乳腺	9（25.7）
子宫及附件	2（5.7）
骨髓	10（28.6）
浆膜腔（胸膜、腹膜、心包）	17（48.6）
胃肠道	9（25.7）
皮肤与软组织	5（14.3）
中枢神经系统复发指数	
低中危	15（42.9）
高危	20（57.1）

2. 疗效与生存期分析：ORR为88.6％（31/35）、CR率为68.6％（24/35）。亚组分析显示，双表达患者的CR率低于非双表达患者（63.1％对75.0％，*P*＝0.795），具体数据参见表2。中位随访时间为28（12～36）个月，10例患者出现了疾病进展，包括8例双表达患者、2例非双表达患者，其中4例患者死亡，随访期间未观察到中枢神经系统复发或进展。随访期内、2年PFS率和OS率分别为68.6％（95％ *CI* 54.0％～87.2％）和87.5％（95％ *CI* 76.7％～100％）。亚组分析显示，双表达患者的2年PFS率低于非双表达患者［54.4％（95％ *CI* 35.4％～84.2％）对85.2％（95％ *CI* 68.3％～100％），*P*＝0.048］。这一发现提示，对于具有双表达特征和高IPI评分的患者，需要采取更为积极的治疗方案以改善其预后。

**表2:** 35例非生发中心起源的弥漫大B细胞淋巴瘤患者的疗效分析［例（％）］

指标	总体（35例）	非双表达（16例）	双表达（19例）	*χ*^2^值	*P*值
ORR（％）	88.6	100	78.9	0.058	0.805
CR［例（％）］	24（68.6）	12（75.0）	12（63.1）	0.149	0.795
PR［例（％）］	7（20.0）	4（25.0）	3（15.8）	0.065	0.691
2年PFS率（％）	68.6（95％ *CI* 54.0～87.2）	85.2（95％ *CI* 68.3～100）	54.4（95％ *CI* 35.4～84.2）	3.910	0.048
2年OS率（％）	87.5（95％ *CI* 76.7～100）	93.7（95％ *CI* 82.6～100）	82.0（95％ *CI* 65.2～100）	0.860	0.350

**注** ORR：总体反应率；CR：完全缓解；PR：部分缓解；PFS：无进展生存；OS：总生存

3. 安全性：12例（34.3％）患者发生3～4级中性粒细胞减少症，5例（14.3％）出现中性粒细胞减少伴发热，3例（8.6％）发生肺炎，其中2例（5.7％）年龄>60岁合并肺部感染的患者，后续单纯给予R-CHOP方案治疗，停用奥布替尼；1例（2.9％）出现了心房扑动，该例患者行射频消融术后继续完成后续治疗。尽管存在上述不良反应，但所有不良反应均可控，未观察到与治疗直接相关的死亡病例。

## 讨论

DLBCL作为最常见的非霍奇金淋巴瘤类型，占B细胞非霍奇金淋巴瘤的30％～40％[6]。当前，R-CHOP方案被视为DLBCL患者的一线标准治疗，尽管其长期生存率可达60％～70％，但仍有30％～40％的患者面临难治或复发，这部分患者的预后不佳[7]–[8]。如何前瞻性鉴别出这些预后不良的患者，并在R-CHOP方案基础上探索联合其他药物治疗，成为当前临床研究的热点与挑战。

在预后评价方面，IPI、COO及分子亚型是常用的评估工具。其中，IPI为评估DLBCL预后的有效手段，高IPI评分的患者在接受R-CHOP免疫化疗后预后较差，IPI评分为3～5分的患者5年生存率约60％[1]。基于免疫组化检测的COO起源，DLBCL可分为GCB和non-GCB两种亚型，non-GCB亚型的预后通常被认为比GCB亚型差[9]。尽管过去有研究尝试将R-CHOP方案与其他药物（如硼替佐米、来那度胺和伊布替尼等）联合用于治疗non-GCB DLBCL，但均未取得成功[10]–[12]。最近的POLARIX研究[13]显示，对于IPI评分为2～5分的DLBCL患者，Pola-R-CHP方案相较于R-CHOP方案在PFS方面具有优势，但OS的改善并不明显，这可能与该研究纳入了部分预后良好的患者有关（例如IPI评分为2分的患者）。因此，如何提前筛选出预后不良的患者进行临床研究，仍是当前面临的主要挑战。

在复发或难治性non-GCB DLBCL中，伊布替尼单药治疗显示出一定的疗效[14]。PHOENIX研究是首个探索布鲁顿酪氨酸激酶抑制剂（BTKi）伊布替尼联合R-CHOP方案在non-GCB DLBCL患者中一线治疗效果的临床研究，研究未达到主要研究终点，分析提示伊布替尼药物不良反应导致的治疗不完整是研究失败的主要原因[12]。但亚组分析显示年轻患者（<60岁）的无事件生存（EFS）和OS得到改善[12]，特别是MCD和N1亚型的年轻患者，在接受伊布替尼联合R-CHOP治疗后，3年EFS率达到100％[15]。此外该研究中BCL2/MYC双表达的患者中，伊布替尼联合R-CHOP方案治疗组相较于仅接受R-CHOP方案治疗组，显著改善了EFS，OS也有改善趋势[16]。上述亚组分析结果均提示预后不良的患者更能从伊布替尼治疗中获益。我们注意到PHOENIX研究的入组患者中，56％左右的患者IPI评分为1～2分，这些患者R-CHOP方案治疗预后较好，再加入BTKi对这部分患者可能获益不大，入组人群不够精准是导致研究未达到主要研究终点的重要原因。

结外累及多部位的DLBCL患者常表现出高侵袭性临床特征，且分子亚型多为预后不良的MCD亚型[2]，这部分患者有可能是BTKi治疗的优势获益人群，由于检测可及性的原因，本研究大部分患者未行二代测序进行基因突变分析。结合分子亚型进一步探索适合BTKi治疗的优势人群是未来研究的方向。

奥布替尼作为一种新型的BTKi，具有低脱靶效应和良好的患者耐受性[17]。本研究将奥布替尼与R-CHOP方案联合用于治疗新诊断的、具有2个及以上淋巴结外病变且IPI评分较高（3～5分）的non-GCB DLBCL患者。结果显示，ORR为88.6％、CR率为68.6％，在中位随访28（12～36）个月后，2年PFS率和OS率分别为68.6％和87.5％。尽管样本量有限，但这些数据提示患者呈现获益趋势。亚组分析进一步显示，双表达组患者的CR率低于非双表达组（*P*＝0.048）。与PHOENIX研究相比，本研究中观察到的严重不良事件较少，且均通过治疗完全恢复，未发生治疗相关死亡事件。未来有必要开展更大规模的临床研究以进一步验证这一联合治疗方案的有效性和安全性。
